# Identification of a Novel Nuclear Localization Signal Sequence in *Chlamydia trachomatis*-Secreted Hypothetical Protein CT311

**DOI:** 10.1371/journal.pone.0064529

**Published:** 2013-05-23

**Authors:** Lei Lei, Xiaohua Dong, Zhongyu Li, Guangming Zhong

**Affiliations:** 1 Department of Microbiology and Immunology, University of Texas Health Science Center at San Antonio, San Antonio, Texas, United States of America; 2 Department of Pharmacology, School of Pharmacy, Hebei Northern University, Zhangjiakou, Hebei, P. R. China; 3 Department of Microbiology, University of South China, Hengyang, Hunan, P. R. China; University of California, San Francisco, University of California, Berkeley, and the Children's Hospital Oakland Research Institute, United States of America

## Abstract

We previously reported that *Chlamydia trachomatis* hypothetical protein CT311 was secreted out of chlamydial inclusion and into host cell cytosol. We now found that CT311 further entered host cell nucleus at the late stage of infection and continued to accumulate in the nucleus of *C. trachomatis*-infected cells. When CT311 was expressed via a transgene in mammalian cells, CT311 protein was exclusively detected in the nucleus, suggesting that CT311 by itself is sufficient for nuclear targeting. However, preexisting nuclear CT311 did not affect subsequent chlamydial infection. Using deletion constructs, we mapped a nuclear localization signal sequence of CT311 to residues 21 to 63 (^21^AVEGKPLSRAAQLRERRKDLHVSGKPSPRYALKKRALEAKKNK^63^). This sequence was sufficient for targeting a heterologous protein into mammalian cell nucleus and it contains two independent clusters of basic residues (^34^RERRK^38^ and ^53^KKRALEAKKNK^63^ respectively). Deletion or alanine substitution of the basic residues in either cluster led to loss of nuclear targeting activity, suggesting that both clusters are critical for the nuclear targeting function. These observations have demonstrated that the hypothetical protein CT311 possesses a novel nuclear localization signal sequence with dual modules of basic residues for targeting host cell nucleus during *Chlamydia trachomatis* infection.

## Introduction

*Chlamydia trachomatis* are Gram-negative, obligate intracellular bacterial pathogens that primarily infect the epithelium of conjunctiva and the genital tract in humans [1]. Despite varying tissue tropism, all *C. trachomatis* organisms share a common bi-phase replication cycle. The *C. trachomatis* organisms invade epithelial cells in the form of elementary bodies (EBs). The intracellular EBs differentiate into reticulate bodies (RBs) that become metabolically active and are able to proliferate. The intracellular life of *C. trachomatis* organisms occurs strictly inside cytoplasmic vacuoles (termed inclusions). The progeny RBs inside inclusions differentiate back into EBs that exit the infected cells and spread to new cells. It is thought that both the intracellular replication and cell to cell spreading significantly contribute to chlamydial pathogenicity [2]–[4]. During intracellular replication, the intra-inclusion chlamydial organisms are found to secrete numerous proteins into the host cells [5]–[14]. These secreted proteins have been hypothesized to both aid in chlamydial intravacuolar replication and facilitate chlamydial spreading. However, the precise functions of these secreted proteins, including CT311 [11], remained unknown. Biochemical characterization including the sub-cellular localization studies may shed new light on the potential roles of these secreted proteins in chlamydial pathogenesis.

Nuclear targeting of bacterial proteins has emerged as a pathogenic mechanism whereby bacterial proteins can directly interact with nuclear molecules or disturb signal transduction pathways, which may result in host cell pathology [15]. For example, the obligate intracellular bacteria *Anaplasma phagocytophilum* organisms translocated an ankyrin repeat-containing protein (AnkA) to the nucleus of infected neutrophils to form DNA-protein complexes [16] while *Ehrlichia chaffeensis* organisms, another obligate intracellular pathogen, secrete an AnkA into host cell nucleus for interacting with an adenine-rich motif to impact expression of genes associated with metabolic and molecular processes and cell structure [17]. The *C. trachomatis* hypothetical CT621 & 622 were also partially detected in the nucleus of *C. trachomatis*-infected cells although neither the mechanisms nor the consequences of CT621 & 622 nuclear localization are known [6], [9]. Another *C. trachomatis* hypothetical protein CT737 was recently reported to partially associate with host cell chromatin although evidence of direct visualization of CT737 in the infected cell nucleus remained lacking [18]. CT737 was designated by the authors as chlamydial nuclear effector (NUE) since it functioned as a histone methyltransferase in a cell-free methylation assay [18]. However, the potential biological significance of these nucleus-localized proteins remains unknown. Nevertheless, the above findings suggest that *Chlamydia trachomatis* organisms have evolved the ability to target host cell nuclei. As the more chlamydial proteins that enter host cell nuclei are identified and characterized, more knowledge will be gained for understanding how *C. trachomatis* organisms can manipulate host nucleus for benefiting chlamydial survival and exacerbating pathology.

Most proteins undergoing nuclear translocation contain a nuclear localization sequence (NLS) [19]. NLS can be categorized into three types based on the distribution of basic residue-enriched motif sequences. The first type is a monopartite NLS consisting of a continuous stretch of basic residues as exemplified by the peptide of “PP**KKKRK**V” in SV large T antigen [20]; The second is a bipartite NLS consisting of two clusters of basic residues separated by 10–12 amino acids as exemplified by the peptide of **KR**PAATKKAGQA**KKKK** in nucleoplasmin [21]; The third is non-classical NLS that do not contain well-conserved sequence [22]. The nuclear localization sequences are normally recognized by nuclear transport proteins and transported to the nucleus through the nuclear pore complex (NPC) [23].

We previously reported that the *C. trachomatis* hypothetical protein CT311 was secreted into host cell cytosol, possibly through a sec-dependent pathway [11]. CT311 is a Chlamydia-specific hypothetical protein. Although bioinformatics analyses failed to reveal any known conserved domains in CT311, CT311 is highly conserved among all chlamydial genomes sequenced so far, suggesting an essential role of CT311 in maintaining chlamydial intracellular parasitism. We now report that CT311 was not only secreted into host cell cytosol but also further translocated to host nucleus during chlamydial infection. A nuclear localization signal sequence consisting of two independent clusters of basic residues was identified in CT311. The two clusters were intervened by 14 amino acids and the basic amino acids in both clusters were required for the nuclear targeting function. These observations have provided important molecular information for further understanding the role of CT311 in chlamydial pathogenesis.

## Materials and Methods

### 1. Chlamydial infection

The *C. trachomatis* L2/LGV-434/Bu organisms were propagated, purified, aliquoted and stored as described previously [24], [25]. HeLa cells (human cervical carcinoma epithelial cells, ATCC cat# CCL2, Manassas, VA) were grown in either 24 well plates with coverslips or tissue culture flasks containing DMEM (GIBCO BRL, Rockville, MD) with 10% fetal calf serum (GIBCO BRL) at 37°C in an incubator supplied with 5% CO2. These cells were inoculated with chlamydial organisms at the appropriate MOIs as indicated in individual experiments. The infected cultures were processed at various time points after infection for immunofluorescence analyses as described below. In some experiments, HeLa cells were transduced with pLenti vectors expressing fusion proteins before chlamydial infection. At 40h after infection with *C. trachomatis* L2 organisms, the cell samples were processed for immunofluorescence assay. A total of 100 transduction positive or negative cells were counted for chlamydial infection. The chlamydial infection rates were compared between transfection positive and negative cells.

### 2. Expression of CT311 and its fragments as V5 or GFP tagged fusion proteins in mammalian cells

To express CT311 and its fragments as V5 tagged fusion proteins, the DNA sequences coding for CT311 and its fragments were cloned into pLenti6.3/V5 vector (Invitrogen, Grand Island, NY) using following primers: for CT311 full length (codon 1-236), forward primer 5′- CGC-GGATCC(BamHI)–ATGAAAAGAGTTATCCTCTGCT-3′, backward primer 5′- CCG-CTCGAG(XhoI)-CGTTTTCCATTTTGCAGATCTTTC-3′; fragment 21-236, forward primer 5′- CGC-GGATCC(BamHI)-GACATGG CCGTAGAAGGAAAGCCTC, backward primer 5′- CCG-CTCGAG(XhoI)- CG TTTTCCATTTTGCAGATCTTTC-3′; fragment 21-129 (codon 21-129), forward primer 5′- CGC-GGATCC(BamHI)- GACATGGCCGTAGAAGGAAA GCCTC-3′, backward primer 5′-CCG-CTCGAG(XhoI)- CGCTCAA TATAGC CATCTTCAGTT-3′; fragment 21-200 (codon 21-200), forward primer 5′- CGC-GGATCC (BamHI)–GACATGGCCGTAGAAGGAAAGCCTC-3′, backward primer 5′-CCG-CTCGAG(XhoI)-CGGGAGACGTTGGGATAGTC AT-3′; fragment 21-63 (codon 21-63), forward primer 5′-CGC- GGATCC(BamHI)–GACATGGCCGTAGAAGGAAAGCCTC-3′, backward primer 5′-CCG-CTCGAG(XhoI)-CGCTTATTTTTTTTAGCTTCTAAAGC-3′. To express CT311 fragments as GFP fusion proteins, the CT311 fragment DNA sequences were cloned into pLEGFP vector (BD biosciences, San Jose, CA) using following primers: fragment 21-236, forward primer 5′-CCG-CTCGAG(XhoI) -ATGGCCGTAGAAGGAAAGCCTC-3′, backward primer 5′-CGC-GGA TCC(BamHI)-CGTTTTCCATTTTGCAGATCTTTC-3′; fragment 21-63, forward primer 5′-CCG-CTCGAG (XhoI)-ATGGCCGTAGAAGGAAAGCCTC-3′, backward primer 5′-CGC-GGATCC(BamHI)-CGCTTATTTTTTTTAGCTTCTAAAGC-3′; fragment 64-236, forward primer 5′-CCG-CTCGAG(XhoI) –ATGCCTTCTATTAGCTGGATAACC-3′, backward primer 5′-CGC-GGA TCC(BamHI)-CGTTTTCCATTTTGCAGATCTTTC-3′; fragment 21-55, forward primer forward primer 5′-CCG-CTCGAG(XhoI)–ATGGCCGTAGAAGGAAA GCCTC-3′, backward primer 5′-CGC-GGATCC(BamHI)-CGACGTTTTTTCAA AGCATAACGA-3′. Site-directed mutagenesis was conducted using a QuikChange XL site-directed mutagenesis kit (Stratagene, La Jolla, CA) according to the manufacturer’s instruction. The basic amino acids were mutated to alanine. The mutations were verified by DNA sequencing. The recombinant plasmids were extracted using QIAprep Spin MiniPrep kit (QIAGEN, Valencia, CA). The purified plasmids were transfected into into HeLa cells using Lipofectamin 2000^™^ (Invitrogen) following manufacturer’s instruction.

### 3. Immunofluorescence assay

The immunofluorescence assays were carried out as described previously [26]. Briefly, HeLa cells grown on coverslips in 24 well plates with or without *C. trachomatis* infection or plasmid transfection were fixed with 2% paraformaldehyde (Sigma, St. Luis, MO) dissolved in PBS (phosphate-buffer saline solution, pH 7.6) for 30 min at room temperature, followed by permeabilization with 2% saponin (Sigma) for an additional 30 min. After washing and blocking, the cell samples were subjected to antibody and chemical staining. The primary antibodies used in this study including: mouse anti-CT311 monoclonal antibody (mAb) clone 7C10 (IgG2a; ref: [11], mouse anti-CPAF mAb clone 100a (IgG1; ref: [14], rabbit anti-Nup153 (cat#HPA-027896, Sigma), rabbit anti-V5 tag (cat# GTX117997, Genetex, Irvine, CA) and rabbit anti-chlamydial organisms (R1L2, unpublished data). The primary antibody binding was visualized using goat anti-mouse or rabbit IgG antibodies conjugated with Cy3 (red) or Cy2 (green) (Jackson ImmunoResearch, city, state). Hoechst (blue, Sigma) was used to visualize DNA. The immunofluorescence images were acquired using an Olympus AX-70 fluorescence microscope equipped with multiple filter sets and Simple PCI imaging software (Olympus, Melville, NY) as described previously.The images were processed using Adobe Photoshop (Adobe Systems, San Jose, CA). The GFP signal was semi-quantitated using the software ImageJ (http://rsbweb.nih.gov/ij/download.html). The fluorescence intensity in the nucleus versus in the entire cell was measured in each cell separately and the % of intensity in the nucleus was calculated for each cell. A total of 10 cells were measured for each sample and the means were used to compare between different samples.

### 4. Statistics

For comparing the GFP fluorescence intensity between different samples, the Wilcoxon rank sum was used. For comparing the infection rate, the Krustal-Wallis was used.

## Results

### 1. The Chlamydia trachomatis hypothetical protein CT311 is translocated into host cell nucleus during the late stage of intracellular infection

**Figure 1:**
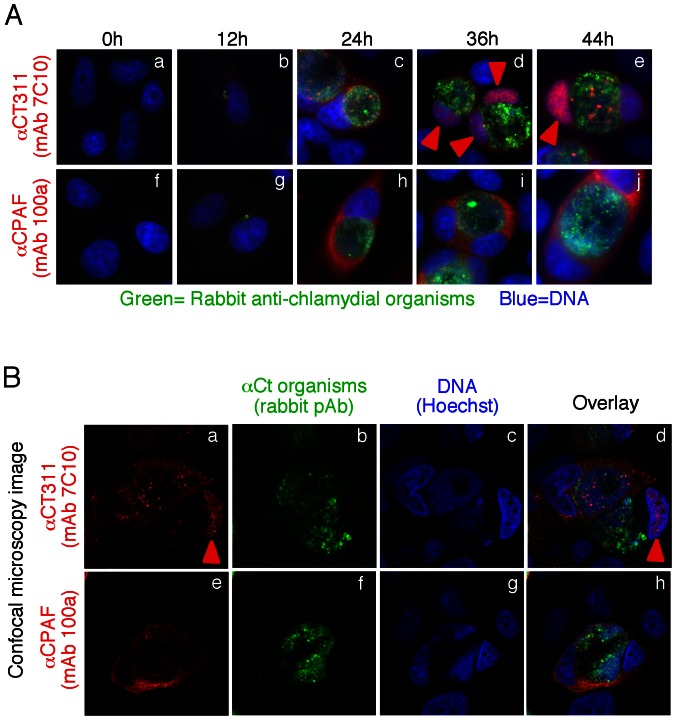
Nuclear localization of CT311 in *C.* *trachomatis*-infected cells. (A) HeLa cells infected with *C. trachomatis* organisms were processed at different time points after infection (as indicated on top of the image) for immunofluorescence labeling of chlamydial organisms (green), CT311 (panels a to e) or CPAF (panels f to j, both red) or DNA (blue). Red arrowheads indicate CT311 localized in the host cell nuclei in samples harvested at 36h (d) and 44h (e) post infection. The images were acquired using a conventional fluorescence microscope. (B) The 36h sample was further observed under a confocal microscope. Red arrowhead indicates nuclear localization of CT311.

**Figure 2:**
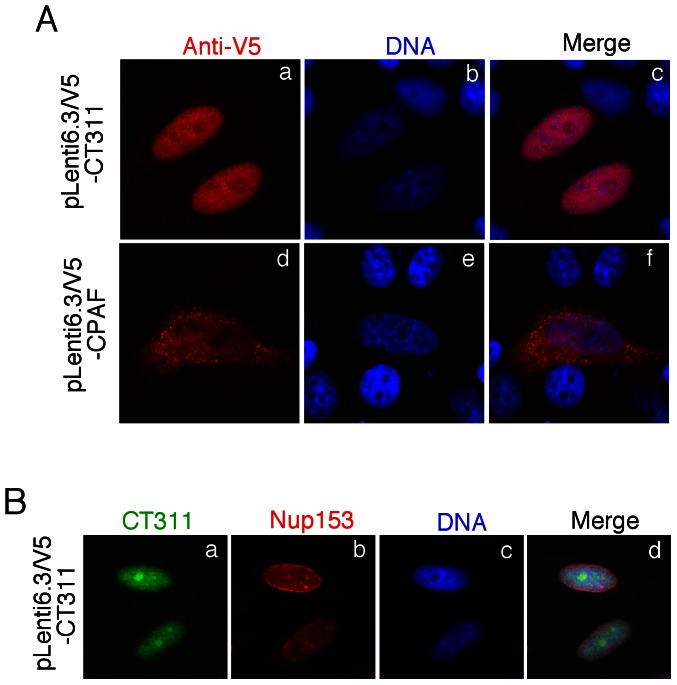
Localization of CT311 expressed via a transgene in mammalian cell nuclei. (A) HeLa cells transfected with pLenti6.3/V5-CT311 (panels a–c) or pLenti6.3/V5-CPAF (panels d to f) were processed 24h after transfection for immunofluorescence labeling of CT311 or CPAF using an anti-V5 tag antibody (red) and DNA using Hoechst dye (blue). CT311 but not CPAF localized in host cell nuclei. (B) The nuclear localization of CT311 was confirmed by co-labeling CT311 (green) and Nup153 (red), a nuclear protein.

We previously reported that CT311 was secreted out of the chlamydial inclusion and into host cell cytosol [11]. Careful analyses of the *Chlamydia trachomatis*-infected cells along the entire infection time course further revealed that most cytosolic CT311 was localized in the host nucleus by 36h after infection and continued to accumulate in the nuclei until lysis of the infected cells (Fig. 1). The CT311 nuclear localization at the late stage of infection seemed to be specific since CPAF, another protein secreted into host cell cytosol by *Chlamydia trachomatis*, was never detected in host cell nucleus at any time after infection. The CT311 nuclear localization was validated in Chlamydia-infected cells using a confocal microscope (Fig. 1B) and recapitulated in CT311 gene-transfected mammalian cells in the absence of infection (Fig. 2A). As expected, expression of CPAF via a transgene in mammalian cells did not result in CPAF localization in the nucleus. The nuclear localization of the transgene-encoded CT311 was confirmed by co-labeling CT311 with the nuclear protein Nup153 (Fig. 2B). These observations suggest that CT311 may contain a nuclear localization sequence.

### 2. Mapping CT311 nuclear localization signal sequence

**Figure 3:**
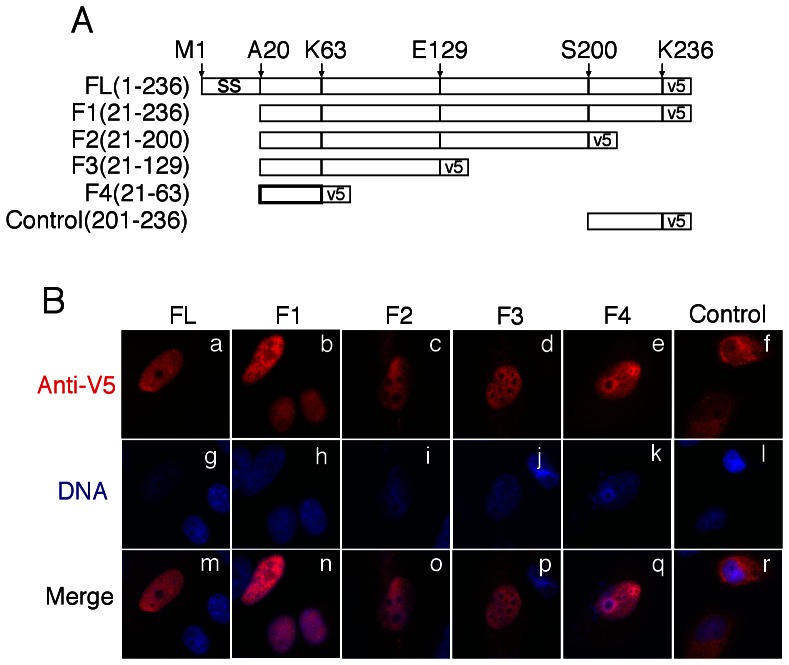
Mapping nuclear localization domain of CT311 to amino acids 21 to 63. (A) Schematic representation of CT311 deletion fragments with a V5 tag fused to C- termini. (B) HeLa cells transfected with pLenti6.3/V5 expressing various CT311 fragments listed in (A) were processed 24h after transfection for immunofluorescence labeling of CT311 using an anti-V5 tag antibody (red) and DNA using Hoechst dye (blue). All CT311 fragments that contain the sequence from residues 21 to 63, including the 21–63 fragment alone (F4), were localized in host cell nuclei. However, the control fragment covering residues 201 to 236 was mainly localized in the cytosol of host cells.

**Figure 4:**
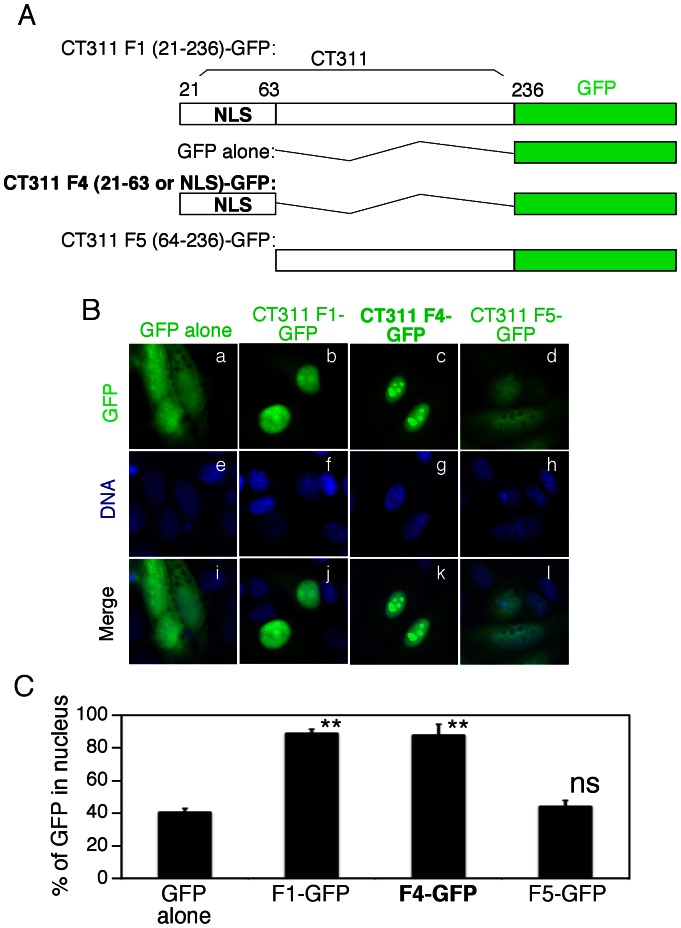
Targeting GFP into nuclei by CT311 fragment 4 (21–63). (A) Schematic representation of various CT311 fragments with the green fluorescence protein (GFP) tagged to the C- termini. The CT311 fragment 4 (21–63) was designated as the nuclear localization signal sequence (NLS) of CT311. (B) HeLa cells transfected with pLEGFP plasmid expressing various CT311 fragments listed in (A) were processed 24h after transfection for labeling DNA using Hoechst dye (blue). The slides were observed under a fluorescence microscope. Both CT311 F1 (panels b & j) and F4 (c & k) but not F5 (d & l) targeted GFP (green) into host cell nuclei. (C) The GFP signal in the nuclei and entire cells was semi-quantitatively measured (from 10 representative cells of each sample) using ImageJ software and the % of nuclear GFP signal was used to compare between cell samples transfected with plasmid coding for GFP alone and those transfected with plasmids coding for CT311-GFP fusion proteins. ** indicates statistically significant differences (P<0.01) and “ns” stands for no significant difference. The data were from 3 independent experiments.

To identify the region responsible for targeting CT311 into mammalian cell nucleus, we made 5 deletion fragments (Fig. 3A) and these fragments were transiently expressed in HeLa cells for intracellular localization by detecting the fusion tag V5 (Fig. 3B). All fragments, as long as the sequence covering residues 21 to 63 was present, were detected in HeLa cell nucleus, suggesting that residues 21 to 63 were critical for CT311 nuclear localization. This conclusion is further supported by the observation that the control CT311 fragment covering residues #201 to 236 failed to enter nuclei. The fact that fragment 4 (F4) consisting of residues 21 to 63 alone was localized in nucleus suggested that F4 contained nuclear localization signal. We designated this fragment as the nuclear localization signal sequence (NLS) of CT311. We further tested whether the CT311 NLS alone is sufficient for targeting a heterologous protein such as the green fluorescence protein (GFP) into mammalian cell nucleus. As shown in Fig. 4, when GFP alone was expressed in mammalian cells, it diffused everywhere (Fig. 4B, panels a & i). However, when GFP was fused to the C-terminus of CT311 mature protein (resides 21 to 236), the fusion protein was only detected in the nucleus (panels b & j), demonstrating that CT311 full length mature protein is able to target a heterologous protein into mammalian cell nucleus. More interestingly, the CT311 NLS that only contains residues 21 to 63 also very efficiently directed GFP into host cell nucleus (panels c & k). As a negative control, the portion of CT311 covering residues 64 to 236 (fragment 5 or F5) failed to significantly alter the intracellular distribution of GFP. The GFP signal was further semi-quantitatively measured and % of GFP signal in the nuclei was compared (Fig. 4C). We found that >90% of GFP signal was in the nuclei of cells expressing GFP fused to CT311 fragments that contain the CT311 NLS but only ∼50% in the nuclei of cells expressing GFP alone or GFP fused to a CT311 fragment that lacks NLS. Apparently, we have been able to demonstrate that the CT311 NLS is both necessary and sufficient for targeting cytosolic GFP into nuclei, despite the high background level of GFP in the nuclei.

### 3. Two clusters of basic residues are required for nuclear targeting of CT311 NLS

**Figure 5:**
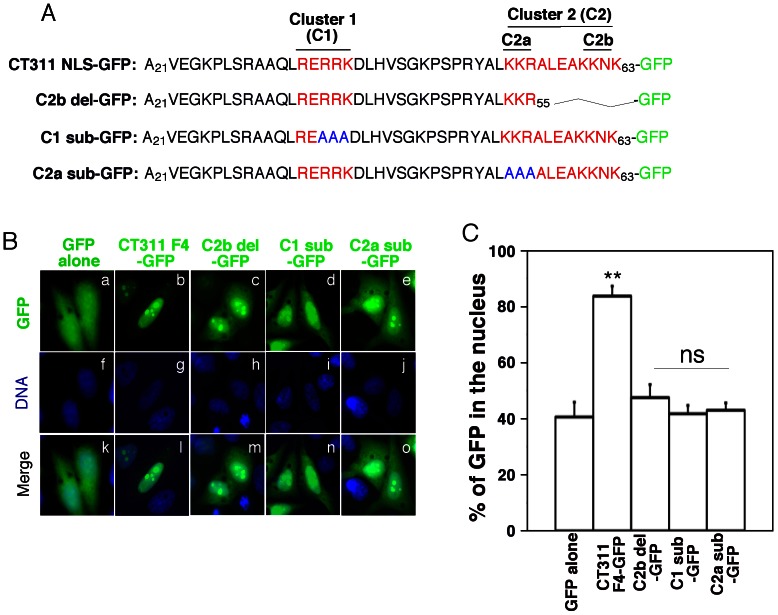
Basic amino acids required for targeting GFP into nucleus. (A) The amino acid sequence covering residue 21 to 63 defined as CT311 fragment 4 (F4) or nuclear localization signal sequence (NLS) was shown with two clusters of basic residues highlighted in red and marked as Clusters 1 & 2 (C1 & C2) respectively. Cluster 2 consists of 2 separate basic residue sub-clusters, designated as C2a & C2b respectively. The sequences of constructs with C2b deletion (C2b del-GFP) and C1 or C2a substitution mutations (C1 sub-GFP or C2a sub-GFP) were also listed. (B) HeLa cells transfected with pLEGFP plasmid alone or expressing the various constructs listed in (A) were processed and observed under a fluorescence microscope as described in Fig. 4B legend. Deletion or substitution of C2b, C1 or C2a effectively blocked the nuclear targeting of GFP by the CT311 NLS. (C) The GFP signal in the nuclei and entire cells was semi-quantitatively measured and the % of nuclear GFP signal from each sample was compared between cell samples as described in the legend of Fig4. ** indicates statistically significant differences (P<0.01) and “ns” stands for no significant difference. The data were from 3 independent experiments.

Amino acid sequence analyses of the CT311 NLS revealed two independent clusters of basic residues, designated as clusters 1 & 2 respectively. As shown in Fig. 5A, cluster 1 consists of ^34^RERRK^38^ while cluster 2 consists of ^53^KKRALEAKKNK^63^. Cluster 2 contains two segregate stretches of basic residues including ^53^KKR^55^ designated as sub-cluster 2a and ^60^KKNK^63^ as sub-cluster 2b. To test whether any of the basic residue clusters or sub-clusters contributes to the nuclear targeting function of CT311 NLS, we made deletion or alanine substitution mutations of these clusters/sub-clusters and evaluated their abilities to target GFP into host cell nucleus. As shown in Fig. 5B, although the CT311 NLS wild type efficiently targeted GFP into host nucleus, none of the mutants was able to do so, which was further validated by the semi-quantitative observation shown in Fig. 5C. These observations and analyses together demonstrated that all clusters/sub-clusters were required for the nuclear targeting function.

### 4. Overexpression of CT311 in the host cell nuclei has no impact on the subsequent infection with *C. trachomatis*

**Figure 6:**
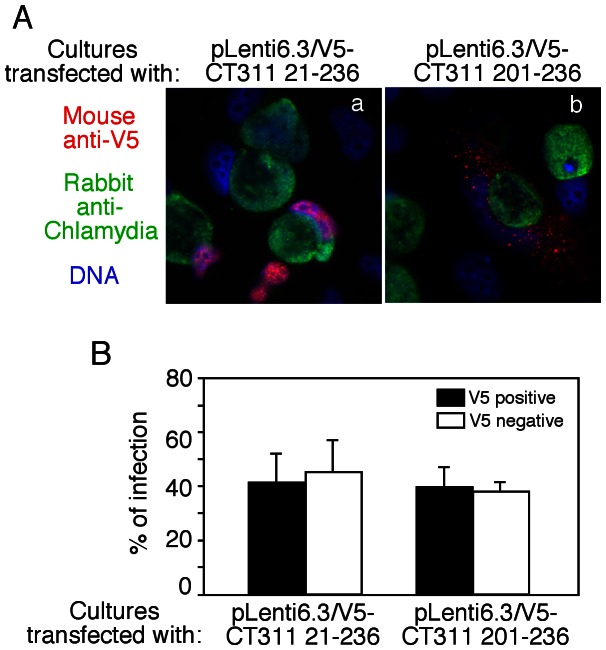
The effect of preexisting CT311 on subsequent chlamydial infection. (A) HeLa cells were transduced with pLenti6.3/V5 vectors expressing either CT311(21-236, panel a) or CT311(201-236, b) fragments. After the transduced cultures were subsequently infected with *C. trachomatis* L2 organisms, both the V5 epitope-positive and -negative cells were counted from the same cultures for the presence of chlamydial inclusions (B). A total of 100 V5 positive (solid bar) or negative (open bar) cells were counted from each sample for the presence of chlamydial inclusions. The chlamydial infection rates as shown along the y-axis were compared between V5 positive and negative cells from the same cultures and there were no statistically significant differences. The data were from 3 independent experiments.

To evaluate whether expression of CT311 in host cell nuclei can affect the subsequent chlamydial infection, we transduced HeLa cells with either pLenti6.3/V5-CT311 21-236 coding for full length mature CT311 protein or plenti6.3/V5-CT311 201-236 lacking CT311 NLS. At 12h after the transformation, the cultures were infected with *C. trachomatis* L2 organisms. When the chlamydial infection rates were compared between V5 tag-labeling positive or negative cells (Fig. 6), we found that the chlamydial infection rates maintained at ∼40% regardless of the cell transduction status and the types of pLenti vector used for the transduction. In addition, the size of the inclusions did not show any significant differences between V5 positive or negative cells either. Thus, we can conclude that the preexisting CT311 in the nuclei of host cells has no effect on the subsequent chlamydial infection.

## Discussion

CT311 is highly conserved hypothetical protein among *Chlamydia trachomatis* organisms, suggesting an important role of CT311 in *Chlamydia trachomatis* biology and pathogenesis. We have now demonstrated that CT311 is first secreted out of the chlamydial inclusion and into host cell cytosol and further translocated to host cell nucleus by 36h after infection. We have presented convincing evidence that the nuclear targeting of CT311 is specific. First, CT311 was detected in nuclei of both *C. trachomatis*-infected cells and CT311 gene-transfected cells while CPAF was only detected in the cytosol but not in the nuclei of either *C. trachomatis*-infected cells or cells transfected with CPAF gene. Second, a NLS was mapped to A21 to K63 of CT311 and the CT311 NLS was sufficient for targeting GFP to host cell nucleus. Third, 3 clusters/subclusters of basic residues were identified in the CT311 NLS and mutating these basic residues completely blocked the nuclear targeting function.

The CT311 NLS is unique. It contains two independent clusters of basic residues intervened by 14 amino acids while a classical bipartite NLS is intervened by only 10 to 12 amino acids [21]. The first cluster is similar to a standard monopartite NLS [19]. However, the second cluster consists of 2 separate sub-clusters of basic residues linked by a 4 non-basic amino acid sequence. Both the number and distribution of basic residue clusters may determine how a NLS is targeted into nucleus. All basic residues in the cluster/sub-clusters seemed to be required for the nuclear targeting function of CT311 NLS (Figure 5 of current study), suggesting that CT311 localization into nucleus is highly regulated. This concept is consistent with the observation that although CT311 is already abundantly secreted into host cell cytosol at the middle stage of infection cycle (24h after infection; Ref: [11]), nuclear localization of CT311 only occurs at the late stage (figure 1 of current study).

Regardless of how CT311 translocation into host cell nuclei is regulated, the most important question is what role the nuclear CT311 may play in chlamydial biology and pathogenesis. Nuclear translocation of CT311 was only detected at the late stage of infection. This is when the chlamydial organisms have completed their intracellular replication and are getting ready to exit the infected cells for spreading to new cells. Can the nuclear CT311 promote chlamydial organism release and spreading? It has been shown that bacterial proteins that enter mammalian cell nucleus can alter cellular functions, including gene activation [27], control of chromatin-regulatory factors [28], modification of nuclear regulators [29], alteration of cell cycle and DNA integrity in the nucleus [30]. The nuclear CT311 may promote chlamydial exiting and spreading by altering host nuclear machineries. Efforts are under way to further investigate the function of CT311 both in the host cell cytosol and nucleus.
